# Metformin as adjunctive therapy in combination with multidrug treatment for multibacillary leprosy: A protocol for a randomized double-blind, controlled Phase 2 trial in Indonesia (MetLep Trial)

**DOI:** 10.12688/wellcomeopenres.19455.1

**Published:** 2023-07-07

**Authors:** Hana Krismawati, Sri V. Muchtar, Mutia Rahardjani, Margareta Oktaviani, Syamsiah, Nelly Imbiri, Dian E. Hasvitasari, Dwi R. Fajrianti, Nico Tarino, Fitri Wulandari, Evelyne Kestelyn, Reinout van Crevel, Stephen L. Walker, Ronald B. Geskus, Annemieke Geluk, Raph L. Hamers, Hardyanto Soebono, Marlous L. Grijsen

**Affiliations:** 1 National Institute of Health Research and Development Papua (NIHRD), Jayapura, Papua, 99351, Indonesia; 2 Department of Dermatology and Venereology, Sandi Karsa Hospital, Makassar, Sulawesi, 90231, Indonesia; 3 Oxford University Clinical Research Unit Indonesia (OUCRU ID), Faculty of Medicine Universitas Indonesia, Jakarta, Indonesia; 4 Oxford University Clinical Research Unit, Ho Chi Minh City, Vietnam; 5 Centre for Tropical Medicine and Global Health, Nuffield Department of Medicine, University of Oxford, Oxford, England, UK; 6 Department of Internal Medicine, Radboud University Medical Center, Nijmegen, 6525 GA, The Netherlands; 7 Clinical Research Department, London School of Hygiene and Tropical Medicine, London, WC1E 7HT, UK; 8 Department of Infectious Diseases, Leiden University Medical Center, Leiden, 2333 ZA, The Netherlands; 9 Department of Dermatology and Venereology, Gadjah Mada University, Yogyakarta, 55281, Indonesia

**Keywords:** leprosy, Morbus Hansen, multibacillary leprosy, leprosy reactions, type 1 reaction, reversal reaction, type 2 reaction, erythema nodosum leprosum, host directed therapy, repurposing drugs, metformin

## Abstract

**Background:**The clinical management of leprosy is complicated by leprosy reactions (LR) causing irreversible nerve damage and disabilities. LR often require long-term use of corticosteroids causing serious side effects. Adjunct host-directed therapy (HDT) is a potentially attractive strategy in leprosy to prevent LR and associated immunopathology, modulate immunological memory that protects against recurrence, and thereby reduce nerve damage, disability and corticosteroid-associated morbidities. Metformin, a well-tolerated, safe and cheap anti-hyperglycaemic drug, is repurposed as HDT in auto-immune and infectious diseases, like tuberculosis (TB). Metformin use in people with diabetes is associated with reduced risks of TB-infection, progression to active TB, treatment failure and TB-mortality. Given the similarities both mycobacteria share, we hypothesize that among persons with multibacillary (MB) leprosy, adjunctive metformin may prevent/mitigate LR.

**Methods:**We will perform a double-blind controlled proof-of-concept trial in which people with newly diagnosed multibacillary leprosy will be randomized (1:1) to metformin hydrochloride 1000mg extended release once daily versus placebo for 24 weeks in addition to standard-of-care WHO MB multidrug therapy (MDT) during 48 weeks. We aim to enrol 166 participants aged between 18 and 65 years, across five clinical sites in two leprosy endemic areas in Indonesia. Primary endpoints are the proportion of participants experiencing a LR and the frequency of (serious) adverse events. Secondary endpoints are the severity and time to first LR, the number of LR, the cumulative corticosteroid usage, and quality of life. The total study follow-up is 48 weeks.

**Discussion:**LR signify the most important cause of irreversible nerve damage leading to anatomical deformities and disabilities, imposing a social and financial burden on those affected. Our study aims to evaluate the efficacy, tolerability and safety of adjunct metformin added to MDT in persons with multibacillary leprosy, and explore its effects on clinical and immunological endpoints.

**ClinicalTrials.gov registration:** NCT05243654 (17/02/2022)

## Introduction

### Background

Leprosy continues to be a public health problem worldwide, with more than 200,000 new cases recorded annually ^1^, predominantly affecting underserved and marginalized populations. Indonesia, a densely populated country in Southeast Asia, has the third highest number of leprosy cases in the world, after India and Brazil ^1^, and has pockets of high endemicity spread throughout the archipelago. In 2019, 17,439 new leprosy cases were reported in Indonesia, of which 86% were multibacillary (MB). Ten of the 34 provinces had a case detection rate of ≥1 per 10,000 population, classifying them as high disease burden areas. 6.4% of new cases had a grade 2 disability at diagnosis, indicative of diagnostic delays, and 11% were children aged 1–14 years, evidence of ongoing transmission in communities ^1, 2^.

### Leprosy reactions

The clinical management of leprosy is complicated by immune-mediated reactions, i.e. unpredictable episodes of exacerbated inflammation occurring before, during or after multi-drug therapy, affecting around 30–50% of people with MB leprosy ^3^. The majority of reactions occur during the first six months after treatment has been initiated ^4– 7^. Leprosy reactions (LR) are the most important cause of irreversible nerve damage leading to anatomical deformities and disabilities, imposing a physical, social and financial burden on people affected by leprosy and their households ^8– 10^. Two distinct LR exist: Type 1 (T1R) or reversal reactions, a delayed hypersensitivity reaction mostly occurring in the borderline forms of leprosy; and Type 2 (T2R), known as erythema nodosum leprosum, which is believed to result from immune-complex deposition and occurs in borderline lepromatous and lepromatous leprosy ^11^. T1R is characterised by acute inflammation and swelling of pre-existing skin lesions, oedema of extremities and face, neuritis, and infrequently systemic symptoms ^12^. T2R is a multisystem disease characterized by the sudden onset of painful, erythematous, subcutaneous skin lesions symmetrically distributed on face and extremities, are not related to the existing leprosy lesions and are usually accompanied by fever, neuritis, arthritis, orchitis, iridocyclitis and nephritis ^13, 14^.

The immunopathogenesis of LR remains poorly understood. The bactericidal effect of MDT creates a reservoir of bacterial breakdown products, which are recognized by the innate immune response and cause migration of pro-inflammatory cytokines and CD4+ T-cells to the site of infection. Activated CD8+ T-cells destroy the *M. leprae*-infected Schwann cells leading to nerve damage. Although the clinical presentation of T1R and T2R are distinct, it is suggested that both reactions share aspects of their immunopathogenesis including enhanced Th1 responses to *M. leprae* and macrophage activation reflected by increased levels of similar pro-inflammatory cytokines, such as IFN-γ and TNF-α,IL-1β and IL-6 ^11^. In T1R, immunological markers like IP-10 and acute phase proteins such as CRP are substantially elevated during the reaction ^11, 15^. In T2R T-cell dysregulation with increased CD4+/CD8+ ratio and reduced numbers of Tregs play a key role, tipping the balance towards pro-inflammation ^16– 18^.

LR are a major clinical challenge as they are often chronic, recurrent and require long-term use of potent immunosuppressant’s. Oral corticosteroids remain the first-line therapy of LR although 40% of people with T1R show no clinical improvement ^5, 19^. Prolonged high dose corticosteroids are often necessary, causing serious adverse effects (SAE, i.e. peptic ulcer, diabetes mellitus, osteoporosis) and even death ^20, 21^. There is a great need to improve treatment strategies and design efficacious, safe and affordable interventions to prevent or limit the development of LR and subsequent neurological impairments and to further elucidate its immunopathogenesis.

### Adjunct host-directed therapy

A new paradigm in management of infectious diseases has emerged that involves therapeutic augmentation of host immune responses and improvement of pathogen eradication, when combined with antimicrobial therapy, and in doing so tilt the host-pathogen equilibrium towards resolution ^22, 23^. In MB leprosy, adjunct host-directed therapy (HDT) could potentially augment antimicrobial efficacy of MDT, prevent LR and associated immunopathology, modulate immunological memory that protects against recurrence of LR, and thereby reduce nerve damage, disability and corticosteroid-associated morbidities. Repurposing drugs, such as metformin, as adjunctive HDT offer a potentially attractive approach that has a biological basis, is safe, affordable and, if effective, could be widely and relatively easily implemented.

### Metformin as a candidate for host-directed therapy

Metformin is a widely used, well-tolerated, cheap oral anti-diabetic drug and is receiving renewed interest for repurposed use as HDT in auto-immune and infectious diseases, like systemic lupus erythematosus (SLE), rheumatoid arthritis and tuberculosis ^22, 24– 29^. Metformin has been associated with reduced cardiovascular events and all-cause mortality, independent of its glycaemic effects, with anti-inflammatory, immunomodulatory, antimicrobial, anti-cancerous, and vasculo-protective effects ^28, 30^. Laboratory studies showed antimicrobial effects of metformin on numerous pathogens, including *Mycobacterium tuberculosis* ( *Mtb*) ^22, 26^, HIV ^31, 32^, hepatitis C ^33^, dengue ^34, 35^ and coronavirus disease 2019 (COVID-19) ^36^. In retrospective cohorts, metformin use in people with diabetes was associated with reduced risks of *Mtb*-infection, progression to active TB, TB treatment failure, recurrent TB, and mortality, even after adjustment for glycaemic control and other anti-diabetic drugs ^37– 41^. In a randomized trial including adults with pulmonary TB, adjunctive metformin diminished excess inflammation reducing lung tissue damage and plasma inflammatory markers ^42^. In *Mtb*-infected mice, use of metformin ameliorated lung pathology, reduced chronic inflammation, and enhanced the specific immune response and *Mtb-*clearance in lungs and spleen by conventional TB-drugs ^22^. 

At the cellular level, metformin has been proposed to enhance host immune response and reduce chronic inflammation through various mechanisms: (i) mice studies demonstrated that metformin reduced intracellular growth of *Mtb* by inducing mitochondrial reactive oxygen species (mROS) production and phagocytosis, through metformin-induced activation of AMPK (adenosine monophosphate–activated protein kinase) and decreased mammalian target of rapamycin (mTOR) signalling ^22, 43^; (ii) Additionally, metformin-induced activation of AMPK stimulates differentiation of T-cells into both regulatory and CD8+ memory T-cells, shifting the balance away from pro-inflammation ^44– 47^. A recent study showed that metabolic reprogramming by metformin empowers CD8+ T-cells and increases the metabolic-fitness to contain *Mtb*-infection ^48^; (iii) Metformin administration in humans and mice also led to major changes in the composition of the gut microbiota, suggesting a possible protective role against inflammatory conditions ^49^.

### Study hypotheses

Based on the phylogenetic and biological resemblance between *Mtb* and *M. leprae* and data from mouse and human studies pointing towards important immunomodulating effects of metformin in *Mtb-*infection, several chronic autoimmune diseases and on metformin’s outstanding safety record, we hypothesize that metformin could be a promising candidate for adjunct HDT in combination with standard MDT for MB leprosy. Metformin could prevent LR, accelerate bacterial clearance and consequently reduce the cumulative dosage of corticosteroids and its accompanied morbidity. Metformin is expected to mitigate nerve damage and disabilities in a predominantly young and working-age population which could improve their quality of life, reduce stigmatization, discrimination and overall health care consumption and costs.

### Study aims

Although the combined available data support the safety and tolerability of concurrent antimicrobial therapy plus metformin, there is a need for a Phase 2, proof-of-concept trial with metformin combined with MDT in MB leprosy to determine its (i) tolerability and safety and (ii) efficacy based on a combination of clinical, immunological and molecular endpoints. This is deemed justified as no suitable animal models for MB leprosy are available to study the drug efficacy against LR.

In parallel, it is important to better characterize the immune and genetic host factors that drive LR in order to design host-directed preventive strategies as well as accurate diagnostic tests to reliably detect or predict the occurrence of LR. As part of the current proposal, we aim to archive prospectively collected blood samples and skin biopsies enabling future in-depth mechanistic studies adopting a system biology approach to elucidate the underlying immunopathology, metformin’s mechanism of action and evaluate novel diagnostics.

This protocol is reported in line with Standard Protocol Items: Recommendations for Interventional Trials (SPIRIT) guidelines ^50^.

## Protocol

### Design and setting

**Figure 1.**
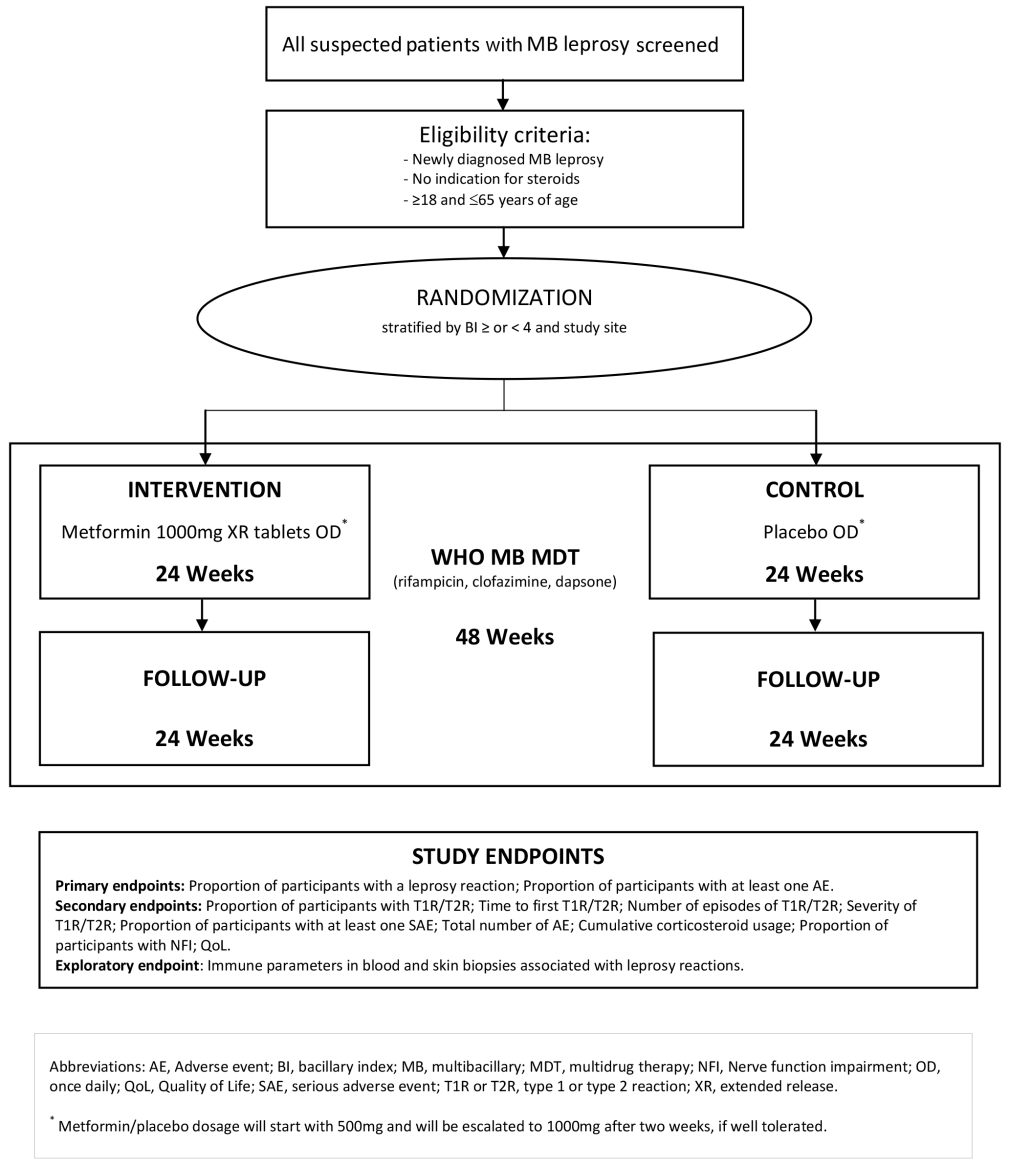
Schematic of study design.

A double-blind, placebo-controlled randomized (1:1) Phase 2 trial evaluating the efficacy, safety and tolerability of adjunct metformin versus placebo for 24 weeks, in addition to standard-of-care MDT (rifampicin 600mg and clofazimine 300mg once a month supervised, 50mg of clofazimine and 100mg of dapsone daily unsupervised) in the management of individuals with newly diagnosed MB leprosy ( Figure 1). The study will take place in five primary health centers (puskesmas) in Jayapura, Papua, and Gowa, Sulawesi, which are areas of high endemicity of leprosy in Indonesia, most of which are MB leprosy. This is protocol version 4.0, dated December 18th 2022.

### Endpoints

***Primary efficacy endpoint***

The proportion of participants experiencing a LR during study follow-up (total 48 weeks).

***Primary safety endpoint***

The proportion of participants with at least one adverse event (AE) within the first 28 weeks of the study.

***Secondary endpoints***

1.   The proportion of participants experiencing a T1R or T2R (as separate endpoints) at 12, 24 and 48 weeks.

2.   Time to first LR and time to first T1R and T2R as separate endpoints, over the full 48 weeks.

3.   The difference in the number of reaction episodes, as well as separately for T1R or T2R.

4.   Severity of T1R and T2R, based on investigator-assessed validated Clinical Severity Scores ^51, 52^.

5.   The proportion of participants with at least one SAE within the first 28 weeks of the trial.

6.   The total number of AE within the first 28 weeks of the trial.

7.   Cumulative corticosteroid usage over the full 48 weeks.

8.   The proportion of participants experiencing clinical nerve function impairment (NFI) developed over the full 48 weeks.

9.   Difference in Quality of Life (QoL) between start and end of treatment intervention, and end of study using validated local language versions of the Dermatology Life Quality Index (DLQI) and the 36-item Short Form Survey (SF-36).

***Exploratory endpoint***

Systemic inflammatory profiling based on circulating inflammatory markers and tissue expression of inflammatory, metabolic, molecular and host immune pathways.

### Case definitions

Leprosy and LR are clinical diagnoses, based on assessment by (an) experienced physician(s) or healthcare worker.

**MB leprosy** is based on the WHO definition of six or more skin lesions, or a positive slit-skin smear with demonstration of *M. leprae*, irrespective of the number of skin lesions ^53^.

A **LR** is defined as the occurrence of T1R or T2R according to below case definitions that may require the initiation or increase in dosage of prednisone/methylprednisolone based on national guidelines. Case definitions that will be used in the study are:

**T1R** characterized by increased inflammation (erythema and oedema) of pre-existing skin lesions and/or neuritis. Participants may have a skin or nerve reaction only or have both at the same time.

- **Neuritis** is defined as spontaneous nerve pain, paraesthesia or tenderness which may or may not be associated with NFI (within the last six months) ^54^.

**T2R** is based on the appearance of crops of tender, erythematous cutaneous and subcutaneous nodules anywhere on the body. Systemic features may be present, but are not obligatory: fever (temperature > 38°C), neuritis, joint and muscle pain, bone tenderness, oedema, malaise, anorexia, lymphadenitis, orchitis, iridocyclitis, uveitis or glomerulonephritis ^51^.

**NFI** is defined as clinically detectable impairment of protective sensory or motor nerve functions that originated within the last six months and necessitates intervention, not necessarily associated with neuritis (silent or asymptomatic neuropathy ^55^).

- **Sensory loss** is defined by a decrease in sensation as measured by Semmes Weinstein monofilament testing. In the hands, this is defined as not being able to perceive the 2g monofilament at 2 points out of 3 sites in each nerve of the hand. In the feet, this is defined as not being able to perceive the 10g monofilament at 3 out of 4 sites of the foot ^54, 56^. The thenar area for the median nerve function, the hypothenar for the ulnar nerve function, and the plantar aspect of the foot for the posterior tibial nerve function will be assessed.
- **Motor loss** is defined by a decrease in voluntary muscle testing score, by 1 point or more from the normal score of 5, using the modified Medical Research Council (MRC) Scale.

### Clinical severity scales

We will use two severity scales that have been prospectively validated in different endemic settings and used in previous clinical trials ^51, 52, 54, 57– 59^:

**T1R:** Modified Clinical Severity Score is used to assess T1R severity. The Score includes 19 items and is expressed as a numeric score (range score 0-48). The maximum score possible for skin (A), sensation (B) and motor function (C) are 9, 9 and 30, respectively. The skin, sensory and motor scores will be summated for each patient per study visit. Outcomes will be assessed by looking at score differences ^59^.

**T1R recurrence or deterioration** is defined as an increase in skin severity score to 4 or more out of 9 AND/OR an increase in NFI defined as worsening of voluntary muscle testing by one point in two or more muscles, or by 2 points in one muscle AND/OR worsening of sensory testing: decreased sensation in at least two out of 3 points per nerve on the hand and/or 3 or more points on the feet. Flare-up is the case when someone is still on steroids.

**T2R:** ENLIST ENL Severity Scale (EESS), including 10 items evaluating multiple organs, and expressed as a numeric score (0 to 30) and classified as mild (≤8) or severe (≥9) ^51^. A change of 5 points on the scale is a meaningful difference. A participant with an increased EESS score of 5 or more points is considered to have deteriorated.

### Inclusion/exclusion criteria

Participants will be considered eligible for enrolment in this trial if they fulfil all the inclusion criteria and none of the exclusion criteria. The trial will enrol participants aged between 18–65 years presenting with newly diagnosed MB leprosy and who have no LR and/or NFI with indication for corticosteroids at the time between diagnosis and trial enrolment. Screened participants who are not eligible will be recorded in a screening log with reasons for screening failure, and will receive local standard-of-care.

***Inclusion criteria***

- Participant is a male or female, aged ≥18 and ≤65 years.
- Participant is newly diagnosed with MB leprosy and receiving MDT ≤28 days.
- Participant is willing and able to give informed consent for participation in the trial.
- Participant is willing to adhere to study follow-up schedule for 48 weeks.

***Exclusion criteria***

- Participant has received MDT >28 days for the current episode of MB leprosy, prior to study enrolment.
- Presence of leprosy reaction and/or nerve function impairment requiring systemic corticosteroids on screening/enrolment evaluation.
- Participants who have been treated for leprosy in the past.
- Chronic systemic corticosteroid use for any other medical condition on screening evaluation (chronic use defined as ≥ 2 weeks).
- History of diabetes mellitus or diabetes mellitus diagnosed on screening evaluation (random blood glucose is elevated ≥200 mg/dL (or ≥11,1 mmol/L) or fasting blood glucose ≥ 126 mg/dL (or ≥7.0 mmol/L)).
- History of hypoglycaemia (random blood glucose <55 mg/dL (or <3.0 mmol/L)).
- History of cardiac failure, ischaemic heart disease, alcoholism, history of lactic acidosis or states associated with lactic acidosis such as shock or pulmonary insufficiency, and conditions associated with hypoxia.
- History of intolerance or hypersensitivity to metformin.
- Estimated glomerular filtration rate (eGFR) ≤30 mL/min/1.73m ^2^ calculated by the CKDEPI equation ^60, 61^.
- ALT/SGPT ≥3 times the upper limit of normal (ULN) on screening evaluation.
- HIV-positive on screening evaluation (screening applicable for sites in Papua only ^a^).
- Female participant in childbearing age who is pregnant (clinically confirmed or urine dipstick for human chorionic gonadotrophin hormone) or breastfeeding.
- Use of metformin within 12 weeks prior to study enrolment.
- Use of other regular hypoglycaemic agents, including insulin.
- Participation in another research trial involving an investigational product within 12 weeks prior to study enrolment.

### Randomisation and treatment allocation

***Randomisation***

All participants who present with newly diagnosed MB leprosy and agree to participate in the trial, will be screened for eligibility. Participants eligible for enrolment, will be randomized to two parallel groups in a 1:1 ratio: metformin for 24 weeks versus identical placebo for 24 weeks. Placebo will be identical in appearance to active drug and dosed and dispensed in the same way. Randomization will be stratified by BI-score (BI ≥ or < 4) and study site. The randomization list will be computer-generated based on random permuted blocks with variable block size. The randomization list will be generated through a statistical code and securely incorporated within the trial database. Based on the randomization list blinded drug packages (treatment packs are labelled with a different code per site) are distsributed to the sites. A 24h web-based randomization service is provided through REDCap. Treatment should start as soon as possible after randomization. Unblinding will be performed after the data analysis is completed or earlier if this is essential for best management of the participant and agreed upon by the Site Principal Investigator (PI) and Chief Investigators. Study medication must be discontinued after unblinding. All instances of unblinding should be reported to the Data Monitoring Committee (DMC).

***Treatment Discontinuation***

The participants may request for any reason to withdraw consent for treatment or the investigator may discontinue treatment for any of the following reasons:

- An AE which requires discontinuation of the investigational medicinal product (IMP, Table 1).
- Pregnancy confirmed by urine dipstick for human chorionic gonadotrophin hormone.
- Request of clinical team if believed the study drug is no longer in the best interest of the patient.

**Table 1.** Overview of adverse events that may require discontinuation of Investigational Medicinal Product.

Adverse events
• Severe acute renal impairment (estimated glomerular filtration rate (30mL/min/1.73m ^2^)
• Lactate ≥ 3 mmol/L and/or onset of metformin-related lactic acidosis (testing triggered in case of clinical suspicion)
• Severe liver involvement: alanine transaminase (ALT) > 3x upper limit normal (ULN), if suspected metformin-associated, or ALT > 4x ULN
• Hypoglycaemic episode (blood sugar < 3.9 mmol/L OR < 70 mg/dL)
• Severe diarrhoea (≥ 5 stools/day with signs of dehydration)
• Mild/moderate diarrhoea, vomiting and/or epigastric pain, if persistent and patient is uncomfortable (despite dose reduction)

### Data collection

Participants will be enrolled in the trial for a total of 48 weeks. Treatment with metformin or placebo (IMP) is the only intervention used in the MetLep Trial. During the first two weeks, adjunctive metformin hydrochloride 500mg extended-release tablets or placebo will be given once daily, escalating to 1000mg metformin hydrochloride extended-release tablets or placebo once daily for another 22 weeks, in addition to standard-of-care MDT. The total duration of IMP intervention will be 24 weeks, while the total duration of MDT will be 48 weeks complying to WHO and national guidelines ( Figure 1). Appropriately trained clinicians and/or nurses with experience in the management of leprosy will be responsible for clinical care. Participants will be reviewed monthly (accumulating to a total of 15 visits spread over 48 weeks) to assess the response to treatment, the development of LR and other outcome measures ( Table 2). Home visits may be used to minimise inconvenience for participants. In case of clinical deterioration (i.e. adverse events, LR), additional unscheduled visits may occur. Study participants who develop a LR will receive appropriate anti-inflammatory treatment according to national guidelines. Participants who miss follow-up visits will be contacted by phone. Clinical and laboratory data (including AEs) will be entered into an electronic REDCap database hosted by OUCRU ID. REDCap is a secure, web-based application designed to support data capture for research providing audit trails for tracking data manipulation.

**Table 2.** Schedule of clinical procedures and laboratory investigations.

	Screening	W0 Enrolment	W2	W4	W8	W12	W16	W20	W24	W28	W32	W36	W40	W44	W48 Completion of MDT	Any time point Leprosy Reaction	TOTAL
**Visit no**	V00	V01	V02	V03	V04	V05	V06	V07	V08	V09	V10	V11	V12	V13	V14	VLR	15
Patient information and informed consent	X																1
Registration Study ID	X																1
Eligibility criteria assessment	X																1
Randomisation		X															1
Initiation metformin/ placebo		X															1
Increase dosage metformin/placebo			X														1
Clinical history and examination																	
Past medical history		X															1
Current signs & symptoms		X	X	X	X	X	X	X	X	X	X	X	X	X	X	X	14
Physical examination ^a^		X	X	X	X	X	X	X	X	X	X	X	X	X	X	X	14
Adverse events assessment			X	X	X	X	X	X	X	X	X	X	X	X	X	X	13
T1R/T2R assessment/ Severity Scale			X	X	X	X	X	X	X	X	X	X	X	X	X	X	13
Quality of life assessment		X				X			X						X	X	4
Clinical Routine Investigations																	
Full blood count	X																1
Serum creatinin	X																1
ALT	X																1
Blood glucose ^b^	X																1
Fingerprick glucose test			X	X	X	X	X	X	X								7
HIV test ^c^	X																1
Pregnancy test (childbearing age women)	X																1
Slit skin smear	X								X						X		3
Research Samples																	
Skin biopsy (6 mm)		X														X	1
Serum (4 mL)		X	X	X		X			X			X			X	X	7
EDTA blood (4 mL)		X		X		X			X							X	4
PAXgene® tube (2.5 mL)		X	X													X	2
Total blood volume for research (mL)		10.5	6.5	8		8			8			4			4	10.5	49

In addition to the scheduled study visits at fixed timepoints (outlined above), additional unscheduled visits may take place in case of a leprosy reaction, adverse events or any other medical complaints and will be recorded as such. a. Physical examination includes body weight, height, blood pressure and nerve function assessment, incl. nerve palpation, sensory testing with five Semmes-Weinstein monofilaments at designated test sites on hands and feet and voluntary muscle power graded using the modified Medical Research Council scale. b. Suspect for diabetes: if random blood glucose is elevated ≥200 mg/dL (or ≥11,1 mmol/L) or fasting blood glucose ≥ 126 mg/dL (or ≥7.0 mmol/L). c. HIV-screening only applicable for participants in Papua.

***Clinical assessments***

During each study visit (some of) the following procedures will be undertaken ( Table 2):

■ Systematic assessments of clinical manifestations and treatment response.
■ Assessment of adherence to IMP (until week 24, W24) and MDT (until W48) through (i) a brief adherence questionnaire; (ii) pill counts on pharmacy records; and (iii) distribution of treatment diaries to each patient by the pharmacist; and (iv) clinical criteria hyperpigmentation and ichthyosis (extensor surfaces).
■ Record all AEs, all SAEs, and all AEs of any grade leading to modification of the IMP or MDT treatment or their interruption or discontinuation during the first 24 weeks when IMP is given. The severity and likely relationship of these AEs to metformin will be assessed and documented by the study team, chief investigators and/or study coordinator.
■ Assessment of LR and its severity using standard case definitions and validated severity scales ^51, 52^.
■ Nerve function assessment is established through nerve palpation, sensory testing and voluntary muscle testing.
■ Assessment of QoL-questionnaires.

***Laboratory assessments***

Full blood count, serum blood glucose, creatinine and ALT will be performed at screening to rule out diabetes mellitus and detect any other relevant abnormalities. Slit skin smears will be performed at W0, W24, and W48 as part of routine clinical care. For participants who have provided consent, a skin biopsy will be performed at W0. From follow-up visit W2 until W24 a fingerprick glucose test will be performed to rule out hypoglycaemia as an additional preventive measure. During any follow-up visits, participants may undergo additional laboratory evaluation for signs or symptoms of potential (S)AEs at discretion of the study team. In case of a LR, a skin biopsy to confirm the diagnosis plus additional blood samples will be performed for bioarchiving.

***On-site biobanking for future in-depth analysis***

The standard participant informed consent form will request consent from participants for genetic analysis as well as biological sample storage for future, ethically approved research. Any proposed plans to use samples other than for those investigations detailed in this protocol will be submitted to the relevant ethics committees prior to any testing. Blood samples (serum and stimulated, heparinized whole blood) and skin biopsies will be stored for future analyses according to the schedule of procedures ( Table 2). Skin biopsies will be used for histopathology, *M. leprae* strain typing, AMR analysis, and, if possible, WGS. Feasibility and range of these additional assays will depend on available funding and emerging scientific insights. The identification and investigation of immunopathogeneis of infection is a rapidly developing research field and novel technologies will likely be identified by the global scientific community during the time period of this study. Therefore, once study samples at serial intervals are available, we will prioritize assays that are most likely to lead to novel insights and patient benefit. Further testing approaches will be driven by advances in diagnostic technologies, such as low complexity multibiomarker tests, genomics, proteomics, metabolomics and/or transcriptomics ^62– 64^.

***Quality control***

Internal and external site monitoring will be carried out at each site to ensure human subject protection, study and laboratory procedures, and data collection procedures meet the international committee of harmonisation (ICH) regulatory guidelines. University of Oxford as the sponsor, represented by the Oxford University Clinical Research Unit Indonesia (OUCRU ID), is responsible to ensure the oversight of the clinical trial conduct and adherence to the protocol. The frequency, type and intensity of routine monitoring and the requirements for triggered monitoring are detailed in the Monitoring Plan. The monitoring will adhere to the principles of ICH good clinical practice (GCP) and the Monitoring Plan. The study may be subject to inspection by the Indonesian drug regulatory authority (BPOM RI) to ensure adherence to GCP and other assessment by relevant institution(s)/authority(ies), e.g. local ethics committee.

### Adverse events and safety reporting

***Tolerability and safety***

Metformin is an inexpensive first-line anti-hyperglycaemic drug for type 2 diabetes mellitus (T2DM). Worldwide, it is the most widely prescribed drug to treat T2DM, and has an excellent safety record. Over the past decades, it has been given to more than 120 million people. Metformin effectively lowers the blood glucose levels by increasing insulin sensitivity and by inhibiting hepatic gluconeogenesis and is not associated with hypoglycaemia in T2DM-patients nor healthy subjects. Previous studies of metformin as adjunct HDT in non-diabetics have used dosages ranging from 1000-2000mg/day ^27, 42, 65– 67^. Metformin offers a favourable tolerability and a high level of patient acceptance. It is commonly used in combination with other drugs because of its beneficial profile. It does not inhibit or induce any CYP-enzyme and is eliminated unchanged in the urine. The trial sponsor has an insurance policy in place in the event of any participant suffering harm as a result of their involvement in the research.

***Adverse events***

The main side-effects of metformin are gastro-intestinal and the risk of lactic acidosis. Approximately 10% of patients report gastrointestinal side-effects, including diarrhoea, cramps, nausea and increased flatulence. Symptoms are typically mild and transient, peaking at the initiation of metformin, and often resolve spontaneously despite continued treatment and dose escalation. Symptoms subside when administered with meals. The reported incidence of metformin-associated lactic acidosis is very low (approximately 0.03 cases/1000 patient-years, with approximately 0.015 fatal cases/1000 patient-years) ^60, 61, 68^. In more than 20,000 patient-years exposure to metformin in clinical trials there were no reports of lactic acidosis. Reported cases occurred primarily in diabetic patients with significant renal insufficiency, often in the setting of multiple concomitant medical/surgical problems and multiple concomitant medications. A Cochrane review of 347 comparative trials and cohort studies with a combined 70,490 patient-years of metformin exposure identified no cases of fatal or non-fatal lactic acidosis ^45^. Metformin does not cause hypoglycaemia in normoglycemic individuals. Rifampicin may enhance the glucose-lowering effect of metformin in non-diabetic individuals by increased hepatic expression of OCT1, resulting in increased metformin delivery to the liver ^69^. Importantly, the combination of rifampicin plus metformin did not reduce baseline blood glucose values or cause hypoglycaemia and rifampicin exerts a countervailing effect by boosting intestinal glucose absorption. We will minimize the risk of AEs in the trial by excluding candidates with a history of renal or liver insufficiency and DM, by counselling at enrolment and follow-up visits regarding the need to maintain adequate caloric intake, and counselling regarding the symptoms of hypoglycaemia and the actions to take if these symptoms occur.

***Dose modifications***

Possible IMP-related AEs will be managed in both randomized groups according to standard clinical practice. Additional requirement will be determined on a case-by-case basis by the attending physician according to the clinical circumstances and guidelines. If treatment is discontinued due to an AE, the investigator will arrange for follow-up visits until the AE has resolved or stabilised. Wherever possible, dose modifications should be performed without unblinding. Participants will not be put at any additional risk by trial randomization, as any participant who develops a suspected adverse drug reaction to the IMP will be managed as if they were receiving metformin.

***Safety reporting***

The principles of ICH GCP apply to this study protocol. All grade 3 or 4, or serious AEs and adverse reactions (ARs), whether expected or not, will be recorded. Non-serious grade 1 or 2 AEs will only be recorded in case they are thought to be related to the IMP or if they result in a change or interruption in MDT. When an AE or AR occurs, the study team and site PI responsible for the care of the participant first assess whether or not the event is serious. If the event is serious and not only related to leprosy, or is fatal, then a SAE form must be completed and the coordinating centre (OUCRU ID) notified within 24 hours. The severity of all AEs and/or ARs (serious and non-serious) in this trial should be graded using the toxicity gradings following Common Toxicity Criteria for Adverse Events CTCAE version 5.0 ^70^.

All AEs will be assessed for seriousness, causality and expectedness. Causality in relation to the IMP includes five categories: unrelated, unlikely, possible, probable, and definitely related. If the causality assessment is unrelated or unlikely to be related, the event is classified as an SAE. If the causality is assessed as possible, probable or definitely related, then the event is classified as an SAR. Expectedness of the AE is assessed using the summary of medicinal product characteristics (SMPC). An unexpected adverse reaction is one not previously reported in the current SMPC at the time the event occurred, or one that is more frequent or more severe than previously reported. If a SAR is assessed as being unexpected, it becomes a suspected unexpected serous adverse reaction (SUSAR).

All applicable SAE/SAR will be reported to the ethics committee and the Indonesian regulatory authority by OUCRU ID based on the requirements stipulated in the Head of BPOM RI Regulation No. 21 year 2015 on Clinical Trial Conduct (Peraturan Kepala Badan Pengawas Obat dan Makanan Republik Indonesia (BPOM RI) tentang Tata Laksana Uji Klinik); and/or relevant local regulations. Any applicable subsequent update to the regulation will be adhered to, respectively. 

The trial will be monitored by an independent Data Monitoring Committee (DMC) who will oversee the safety and progress of the trial and provide recommendations to the Trial Management Group (TMG) and sponsor. The DMC will meet periodically according to the DMC charter and will review the safety and efficacy of IMP after 50% of targeted recruited numbers have been included in the trial. The DMC will have full access to the unblinded data.

### Statistical analysis

***Sample size***

A formal sample size calculation was not conceivable due to lack of preliminary data regarding metformin and leprosy. Because of limitations in feasibility and allocated budget, the target sample size will be 150 participants (75 per arm). To account for up to 10% lost-to-follow-up, the sample size is increased to 166 participants. If we assume that 50% of participants in the standard-of-care (control) arm will develop a T1R/T2R during the 48 weeks study follow-up ^4^, a sample size of 75 participants per arm, achieves 89% power at a 2-sided 0.05 significance level, based on an estimated intervention effect of 50% relative risk reduction.

***Analysis of the primary efficacy and safety endpoints***

The primary analysis will be intention-to-treat containing all randomized participants. In addition, the primary endpoints will be analysed in the per-protocol population, which will exclude participants with a final diagnosis other than MB leprosy or major protocol violations, namely:

- Participants who took <80% of the allocated doses of the study drug during the intervention period of 24 weeks based on the registered pill counts on pharmacy records, and/or
- Participants who missed more than 14 consecutive daily dosages of the study drug during the intervention period of 24 weeks, and/or
- Participants who are lost to follow-up during the initial intervention period of 24 weeks. We assume that loss to follow-up beyond 24 weeks is unrelated to the outcome.

The primary efficacy endpoint is the proportion of participants experiencing a LR, evaluated at week 12, 24 and 48. The effect measure is the treatment difference between the intervention (MDT plus metformin) and control (MDT plus placebo) group. The treatment difference at the three time points will be quantified in a regression model that includes BI and study site as covariables. Proportions will be compared and a logistic regression model will be used per time point. If loss to follow-up is less than 5% in each group, proportions will be compared and a logistic regression model will be used per time point. If loss to follow-up is 5% or more in each group, censored data will be corrected using pseudo-values techniques. The heterogeneity of the treatment effect will be assessed by including an interaction term between treatment and BI, as well as between treatment and study site (two separate models). For the safety endpoint, the proportion of participants with at least one AE within the first 28 weeks will be compared. Depending on the outcome, chi-squared or Fisher’s exact tests will be used to compare the treatment arms. The analyses to be performed will be documented in the Statistical Analysis Plan (SAP), which will be finalized before the database is unlocked and the final analysis with the unblinded data is performed. R software will be used.

***Analysis of the secondary endpoints***

For all outcomes, we make a direct comparison by using treatment arm as the only variable. Secondary endpoints 1 and 5 are analyzed in the same way as the two primary endpoints. For time to first LR, we compute and plot Kaplan-Meier curves by treatment arm and fit Cox proportional hazards models that include BI and study site. Although the value of the hazard ratio cannot be given a causal interpretation, we include this model because it is the standard approach in time-to-event analyses. Endpoints 3 and 6 will be compared via a quasi-Poisson regression model. Severity of T1R and T2R are compared at the start of each episode, as well as their trends over the duration of each episode. We use random effects to take account of the correlation within individuals. Cumulative corticosteroid usage is modelled via linear regression. If needed, we will use the Box-Cox procedure to determine an appropriate transformation and allow for extra values at zero via a zero-inflated model. Clinical NFI is compared via chi-squared or Fisher’s exact test. Quality of life is compared over the four time points using mixed effects models. If the distribution in QoL is too skewed, we will use beta regression using generalized estimating equations to correct for correlation within individuals.

### Ethical considerations

***Consent***

Before any of the trial-related procedures are conducted, the participant or a legally acceptable surrogate (if the participant lacks capacity) must undergo an informed consent procedure in their own language. Participants will be informed about the exact nature of the study, potential side-effects of metformin and MDT, signs and symptoms of LR and NFI and any risks involved in taking part. Participants will be advised to immediately return to the clinic once symptomatic complaints or abnormalities occur. Participants and/or surrogates will be given proficient time to consider participation in the trial and ask questions if needed. The PI is responsible for ensuring that all vulnerable participants are protected and participate voluntarily in an environment free from coercion or undue influence. If consent was provided by a relative, the participant should be consulted and consent recorded if and when they have the capacity to do so. A separate, optional, storage consent will be asked for long term storage of blood and skin samples for future immunogenetic research. Absence of this consent, does not effect trial eligibility.

***Confidentiality***

The trial will be conducted in accordance with the principles of the Declaration of Helsinki. All participant-related information will be kept confidential. Participants will be identified by a unique study identification number that will be used for documentation (i.e. case report forms, IMP labels, samples) and stored in secure locked locations at each site. Electronic data will be stored in password-protected databases of which only study staff have access to. Scientific publications, reports or presentations will never identify participants by name or initials. When the research team reviews clinical notes, they are bound by professional confidentiality. Clinical information will not be released without written permission, except as necessary for monitoring, audit and inspection purposes. Study documentation will be kept in a secure location and held for 15 years after the end of the trial.

***Withdrawal***

Each participant has the right to withdraw from the study at any time. If a participant withdraws from the trial, the medical data collected during their previous consented participation in the trial will be kept and used in the analysis. Participants who stop the trial within 24 hours of enrolment will be replaced. Participants who stop the trial follow-up hereafter will not be replaced, as the total sample size includes adjustments for loss to follow-up.

***Ethical approval***

Ethical approval has been obtained from Universitas Gadjah Mada Research Ethics Committee (KE/FK/0695/EC/2021; 21.06.2021), Observational/Interventions Research Ethics Committee of the London School of Hygiene & Tropical Medicine (26618; 02.03.2022) and Oxford Tropical Research Ethics Committee (14-21; 29.07.2021). Approval has been received from the Indonesian drug regulatory authority (RG.01.06.1.3.04.22.76; 28.04.2022). Additional permissions have been received from the clinical sites and local governments. Future amendments to the study protocol will be approved by above parties before being implemented.

### Data sharing and dissemination

The study is registered at ClinicalTrials.gov (NCT05243654; 17.02.2022) and at Indonesian research registry (NA-ZBMDZ7C; 02.06.2022). OUCRU ID has a data sharing policy in place. Data exchange complies with local Information Governance and Data Security policies. Study results will be shared through presentations at scientific conferences and peer-reviewed journals. The collected data will help build evidence-based guidelines and provide opportunities for dissemination and engagement with local communities, stakeholders and (inter)national policy makers. The sites and collaborators participating in this study have an excellent position to disseminate results and influence practice and policy in and outside of Indonesia. Trial results will be reported in concordance with the CONSORT checklist.

### Trial committees

The trial sponsor is the University of Oxford. The TMG will be formed to oversee the management and progress of the trial. This will include the chief investigator, site PIs, study coordinator, co-investigators, trial statistician, and/or data manager. The group will meet quarterly or more often as required. The DMC will advise the TMG regarding continuation, modification or premature closure of the trial.

### Trial status

The trial is currently enrolling participants. The sites in Jayapura, Papua, started enrolling participants in October 2022 and the sites in South Sulawesi in April 2023.

## Discussion

LR are the most important cause of irreversible nerve damage in persons affected by leprosy, even though the immunopathogenesis is poorly understood. LR are a major clinical challenge as they are often chronic, recurrent and require long-term use of systemic corticosteroids causing (S)AE. Adjunct HDT may have the potential to improve immunological responses and thereby reduce nerve pathology, disability and corticosteroid-associated morbidities. The TRIPOD I trial illustrated that low-dose prophylactic corticosteroids prevented LR and NFI in MB leprosy in the short-term, but had no effect after one year ^71^. Repurposing immunomodulating drugs, such as metformin, as adjunct HDT may offer a potentially attractive approach that has a biological basis, is safe, affordable, widely available and, if effective, could be relatively easily implemented. The MetLep Trial will be the first double-blind randomized clinical trial evaluating the efficacy, tolerability and safety of adjunctive metformin combined with MDT in persons with MB leprosy to mitigate LR and subsequent nerve damage and corticosteroid use and to further elucidate its immunopathogenesis. If Phase 2 trial results are positive, this trial will inform the design of a Phase 3 efficacy trial with extended follow-up.

## Data Availability

No data are associated with this article. Oxford University Research Archive: SPIRIT checklist for ‘Metformin as adjunctive therapy in combination with multidrug treatment for multibacillary leprosy: A protocol for a randomized double-blind, controlled Phase 2 trial in Indonesia (MetLep Trial)’. https://doi.org/10.5287/ora-jbn07qyzd ^50^. Data are available under the terms of the Creative Commons Attribution 4.0 International license (CC-BY 4.0).

## Footnotes

^a^Based on reported HIV prevalence of 2.3% in general population in Papua (source: MOH)
