# Supplementary material for: Metformin as adjunctive therapy in combination with multidrug treatment for multibacillary leprosy: A protocol for a randomized double-blind, controlled Phase 2 trial in Indonesia (MetLep Trial)
Source: Wellcome Open Res. 2024 May 9;8:289. Originally published 2023 Jul 7. [Version 2] doi: 10.12688/wellcomeopenres.19455.2 (PMC11131563; doi:10.12688/wellcomeopenres.19455.2)
Supplement: Peer review history

# Reviewer report: Reviewer response for version 2

**DOI:** 10.21956/wellcomeopenres.23665.r82461

**Published:** 2024-05-23

**Authors:** Veincent Christian F Pepito

**Affiliations:** 1 Ateneo de Manila University, Quezon City, Metro Manila, Philippines

None.

Is the study design appropriate for the research question?

Yes

Is the rationale for, and objectives of, the study clearly described?

Yes

Are sufficient details of the methods provided to allow replication by others?

Yes

Are the datasets clearly presented in a useable and accessible format?

Not applicable

Reviewer Expertise:

Leprosy; Epidemiology

I confirm that I have read this submission and believe that I have an appropriate level of expertise to confirm that it is of an acceptable scientific standard.

# Reviewer report: Reviewer response for version 1

**DOI:** 10.21956/wellcomeopenres.21551.r65574

**Published:** 2023-09-20

**Authors:** Veincent Christian F Pepito

**Affiliations:** 1 Ateneo de Manila University, Quezon City, Metro Manila, Philippines

This is a proposal of a trial to assess the efficacy of metformin on preventing leprosy reactions. The trial is well-designed and well-written, but I have a few comments:

1. Justify use of placebo - are there no other standard treatments in use to merit its use? Also describe what placebo will you be administering, if possible.
2. Since safety is a primary outcome, I doubt if the sample size is sufficient to detect significant differences in rates of AE within the first 28 weeks of the study considering that you only considered the efficacy endpoint in computing for the sample size requirement.
3. Clarify procedure for allocation concealment and blinding. Who will be blind to what?
4. Describe checks to ensure effectiveness of randomization.
5. Since this is a trial and we are moving away from using logistic regression, I suggest that you model risk of experiencing LR and risk of participants with at least one AE instead of its odds. Thus, use alternatives to logistic regression to model risk directly instead of odds. Also control for covariates that could differ between treatment arms despite randomization. 
6. Also describe interactions between metformin and other drugs in the MDT like dapsone or clofazimine, not just with rifampicin. 

Is the study design appropriate for the research question?

Yes

Is the rationale for, and objectives of, the study clearly described?

Yes

Are sufficient details of the methods provided to allow replication by others?

Yes

Are the datasets clearly presented in a useable and accessible format?

Not applicable

Reviewer Expertise:

Leprosy; Epidemiology

I confirm that I have read this submission and believe that I have an appropriate level of expertise to confirm that it is of an acceptable scientific standard, however I have significant reservations, as outlined above.

# Reviewer report: Reviewer response for version 1

**DOI:** 10.21956/wellcomeopenres.21551.r65581

**Published:** 2023-08-30

**Authors:** Roberto Arenas-Guzmán

**Affiliations:** 1 Hospital General "Dr. Manuel Gea González", Mexico City, Mexico

Clinical management of leprosy reactions is critical, because in some cases thalidomide is a good option, but it has many limitations and not always is available, in the other hand corticosteroids causes serious side effects.

 As the proposal metformin, seems to be a good option, because is a well-tolerated, safe and cheap anti-hyperglycaemic drug, and it has been showed an interesting response in auto-immune and infectious diseases, like tuberculosis.

 I think it is a handicap, but if possible, the authors will try to perform in 166 patients, a double-blind controlled and randomized proof-of-concept trial in which people with newly diagnosed multibacillary leprosy to metformin hydrochloride 1000mg extended release once daily versus placebo for 24 weeks in addition to standard-of-care WHO MB multidrug therapy (MDT) during 48 weeks.

 In conclusion this study aims to evaluate the efficacy, tolerability and safety of adjunct metformin added to MDT in persons with multibacillary leprosy, and explore its effects on clinical and immunological endpoints, and at the same time improving nerve damage deformities and disabilities.

Is the study design appropriate for the research question?

Yes

Is the rationale for, and objectives of, the study clearly described?

Yes

Are sufficient details of the methods provided to allow replication by others?

Yes

Are the datasets clearly presented in a useable and accessible format?

Yes

Reviewer Expertise:

Neglected tropical diseases

I confirm that I have read this submission and believe that I have an appropriate level of expertise to confirm that it is of an acceptable scientific standard.
